# A standardized human embryoid body platform for the detection and analysis of teratogens

**DOI:** 10.1371/journal.pone.0171101

**Published:** 2017-02-09

**Authors:** Anthony Flamier, Supriya Singh, Theodore P. Rasmussen

**Affiliations:** 1 Department of Pharmaceutical Sciences, University of Connecticut, Connecticut, United States of America; 2 Department of Molecular and Cell Biology, University of Connecticut, Storrs, Connecticut, United States of America; 3 University of Connecticut Stem Cell Institute, Storrs/Farmington, Connecticut, United States of America; Newcastle University, UNITED KINGDOM

## Abstract

Teratogens are compounds that can induce birth defects upon exposure of the developing fetus. To date, most teratogen studies utilize pregnant rodents to determine compound teratogenicity *in vivo*. However, this is a low throughput approach that cannot easily meet the need for comprehensive high-volume teratogen assessment, a goal of the US Environmental Protection Agency. In addition, rodent and human development differ substantially, and therefore the use of assays using relevant human cells has utility. For these reasons, interest has recently focused on the use of human embryonic stem cells for teratogen assessment. Here we present a highly standardized and quantitative system for the detection and analysis of teratogens that utilizes well-characterized and purified highly pluripotent stem cells. We have devised strategies to mass-produce thousands of uniformly sized spheroids of human ESCs (hESCs) that can be caused to undergo synchronous differentiation to yield embryoid bodies (EBs) in the presence and absence of suspected teratogens. The system uses all human cells and rigorously controlled and standardized EB culture conditions. Furthermore, the approach has been made quantitative by using high-content imaging approaches. Our system offers distinct advantages over earlier EB systems that rely heavily on the use on mouse ESCs and EB aggregates of stochastic sizes. Together, our results show that thousands of suspected teratogens could be assessed using human EB-based approaches.

## Introduction

Annually, 3 to 5% live births in the United States are impacted by birth defects leading to over 100,000 occurrences of birth defects per year [1, 2]. Teratogens include environmental compounds to which women are exposed before or during pregnancy through food, drinking water, airborne sources, and through dermatological exposure. In addition, many pharmaceuticals have teratogenic activities. The U.S. Environmental Protection Agency has a long-standing mission to identify potentially toxic compounds through the Toxcast program which aims to assess thousands of chemicals of potential concern [3]. However, methods to screen vast collections of compounds for teratogenicity are currently laborious and plagued by relatively low throughput, though recent computational approaches have identified a subset of ToxCast compounds, many with potentially teratogenic effects [4].

Teratogens are compounds that cause birth defects, and these can be either pharmaceutical agents to which conceptuses are exposed *in utero*, or environmental compounds. Thalidomide is a sedative used widely form 1957 to 1961 during pregnancy, and over 10,000 infants were born with birth defects after thalidomide exposure in utero [5–8]. These unfortunate individuals presented with limb abnormalities and neural tube closure defects [7]. Sodium valproate is a widely prescribed anti-epileptic and mood stabilizing agent which is also a histone deacetylase inhibitor and known teratogen [9–13]. Though women who may potentially become pregnant are advised to avoid valproate, unintentional exposure may occur during the first days of pregnancy at the zygote, morula, blastocyst, and gastrulation stages prior to detection of pregnancy.

Pharmaceuticals and xenobiotic compounds have been traditionally tested for teratogenicity in pregnant animal models and rodent embryo culture [14], and human cell culture models for teratogenicity are not well developed at present. However, recent innovative approaches include the use of embryonic stem cells to monitor metabolomic changes in response to toxicants [15], and assessments of teratogenic effects in zebrafish, where embryonic development can be monitored by virtue of a transparent fish body plan [16].

Traditional animal assays for teratogens are excellent (since the readout is based on direct teratological impacts on developing embryos), but practical difficulties exist with these approaches. The first impediment is that animal teratological assays are very low throughput. In addition, there is concern that species-specific differences between rodent and human prenatal development impact the interpretation of suspected teratogens. Furthermore, in mammals (including both rodents and humans), both male and female germ line development occurs during embryonic development. For this reason, when dosing a pregnant rodent, the mother, the developing embryo, and the developing embryonic gonadal primordia are simultaneously dosed. Thus, it is not immediately apparent whether the effects of a compound act directly upon the mother, the fetus, or the offspring of the exposed fetus, thus creating a conundrum that requires the assessment of at least 2 generations of offspring for birth defects.

For the reasons above, recent interest has focused on the development of cell culture-based systems to detect teratogens. ESCs can be cultured indefinitely and coaxed to differentiate in vitro into a multitude of terminally-differentiated cell types, thus making them an attractive choice for use in teratology assays. Initial attempts with ESCs relied upon direct exposure of undifferentiated mouse ESCs to potential teratogens followed by assays of differentiation. More recently, approaches have been developed that use ESC aggregates to initiate the formation of embryoid bodies (EBs). Standard methods for producing EBs involve partially disaggregating mouse or human ESC colonies to yield agglomerates of dozens to hundreds of cells, and these aggregates are cultured in media that lack signalling molecules that preserve the pluripotent state (i.e. LIF in the case of mouse ESCs and basic FGF (b-FGF) in the case of human ESCs). Under these conditions, EBs grow and spontaneously differentiate into cellular lineages that contain cells of ectodermal, endodermal, and mesodermal identities [17–21]. Together, these and many other studies show that differentiating EBs come to contain a great many cell types derived from all three of the major germ layers, and that the accumulation of these cells can be monitored by cell biological assays. Furthermore, developing EBs exhibit the induction of a wide array of germ layer-specific gene expression patterns. However, monitoring complex alterations of cellular content and gene expression in EBs developing in the presence of compounds is labor intensive, and EB approaches using uncontrolled aggregation yield EBs of random size and shape, making the assessment of EB growth difficult to quantitate and of low throughput. We aimed to improve the throughput of initial EB teratogen screens by developing standardized systems to produce uniformly sized EBs combined with synchronous differentiation.

Here we present a standardized human EB system for the detection and modeling of teratogen action. Our goals were to develop a highly standardized system, with uniformly-sized human EBs assembled from pristinely pluripotent and quality-controlled hESC cultures. Our aim was to improve the quality and accuracy of a human EB system for the detection of teratogens that could be easily adapted to automated high content imaging solutions, with improved throughput so that in the near future, hundreds to thousands of compounds can be expediently assessed for teratological risk.

## Materials and methods

### Cell culture

H9 hESCs were obtained from WiCell and cultured on hESC-qualified Matrigel (Corning #354277). For all assays, we utilized a highly characterized lot of H9 hESCs curated in the Rasmussen Laboratory established from an initial freeze at passage 19. Media was changed every day with mTeSR1 (Stem Cell technologies #05857) and mechanically passaged every 4–6 days. Embryoid bodies were formed and maintained in EB Aggrewell medium (Stem Cell Technologies #05893). Fibroblast cell lines IMR-90 and IMR-91 (Coriell, #I90-83 and #I91S-04) were grown at 37°C with 5% CO2 on 0.1% gelatin-coated cell culture-treated plates under aseptic conditions. Culture media consisted of 4.5 g/L glucose DMEM (Invitrogen, #10938025) supplemented with 10% FBS (Atlanta Biologicals, #S10250H) and 100X Glutamax (Invitrogen, #35050061).

### Cytotoxicity assays

IMR-90 (I90-83) and IMR-91 (I91S-04) cells were plated on day zero at passages 10 and 14, respectively. Fibroblasts were treated in quadruplicate with 0 mM, 0.1 mM, 1 mM, 10 mM, 100 mM, and 1 M concentrations of VPA, caffeine, and penicillin G dissolved in culture media on day 2. On day 3, cells were measured for viability using an MTS assay (Promega, #G3580) in a SpectraMax Paradigm plate reader from Molecular Devices.

### Flow cytometry

H9 hESCs were incubated with Accutase (Invitrogen, #A11105-01) for 5 minutes or until partial detachment and passed through a 40 μm cell strainer. Single cells were then pelleted and resuspended with fresh 4% paraformaldehyde (PFA). After 10 minutes of incubation at room temperature, cells were exposed to methanol for 15 minutes on ice. Following cell fixation and permeabilization, the cells were incubated with primary antibodies for 30 minutes on ice using Stain buffer (BD Biosciences, #554657): Mouse α-human Tra1-60-FITC (BD Biosciences, #560380), Mouse α-human SSEA4 PE (BD Biosciences, #560128), Mouse α-human Oct3/4 AlexaFluor 647 (BD Biosciences, #560329), Mouse α-human Nanog-PerCP-Cy5.5 (BD Biosciences, #562259).

10,000 events were collected using FACS Calibur instrument (BD Biosciences) after compensation using CompBeads plus (BD Biosciences, #560497). All data were analyzed using FlowJo software.

### Live cell staining

Live stains of H9 colonies were performed using StainAlive α-SSEA-4 antibody (DyLight 550) (Stemgent, #09–0097). Briefly, fresh mTeSR1 media containing 10 μg of StainAlive α-SSEA-4 antibody was applied to cultures and incubated 30 minutes at 37°C. After two washes with fresh warm mTeSR1 media, hESC cultures were analyzed on an inverted fluorescence microscope.

### Magnetic immunoselection of pluripotent cells

Magnetic purification was conducted using a TRA-1-60 selection kit (Miltenyi Biotec, #130-100-832) following manufacturer’s instructions. Briefly, H9 cells at the day of harvest were incubated with Accutase (Invitrogen, #A11105-01) for 5 minutes or until partial detachment, passed through a 40 μm cell strainer and resuspended into fresh medium. 2x10^6^ single cells were then incubated for 5 minutes at 4°C with anti-TRA-1-60 MicroBeads with ROCK inhibitor. Cell-bead conjugates were then collected on magnetic columns under magnetic field and TRA-1-60 positive cells were collected using 1 ml of culture medium.

### Embryoid body formation

Single cell suspensions of TRA-1-60 positive cells were deposited into AggreWell plates (Stem Cell Technologies) and allowed to aggregate into spheroids. Briefly, 1x10^6^ cells positive for TRA-1-60 were suspended in 5 ml of EB Aggrewell medium (Stem Cell technologies #05893) and added into a well of an AggreWell400Ex plate (Stem Cell Technologies, # 27840) containing approximately 4,700 microwells with a diameter of 400 μm and pretreated with AggreWell rinsing solution (Stem Cell Technologies, # 07010). AggreWell plates were centrifuged at 100 x g for 3 minutes to deposit the cells in the microwells and incubated at 37°C for 24 hours, during which time spherical aggregates of deposited pluripotent cells (spheroids) were formed.

### qEB assay

Spheroid aggregates were collected by firmly pipetting in the AggreWell plates and EB cultures were initiated by depositing spheroids into low-adherence culture plates treated with either DMSO (vehicle only), Valproic acid (VPA) (Sigma-Aldrich, #P4543-10G; 1mM), caffeine (Sigma-Aldrich, #C0750-5G; 0.26mM) or penicillin G (Sigma-Aldrich, #P3032-1MU; 2.8mM).

### Gene expression analysis

RNA was extracted using the RNeasy Mini kit (Qiagen, # 74104) according to manufacturer instructions. 1μg of RNA was further converted into complementary DNA using iScript cDNA Synthesis Kit (Bio-Rad, #1708890). PCR reactions were performed using GoTaq green master mix (Promega. #M7112) and loaded on a 1% agarose gel.

Primer sequences are:

Nestin_F: 5’-GAAGGGCAATCACAACAGGT-3’

Nestin_R: 5’-GGGGCCACATCATCTTCCA-3’

B-III tub F: 5’-GGCCAAGGGTCACTACACG-3’

B-III tub R: 5’-GCAGTCGCAGTTTTCACACTC-3’

NEFH F: 5’-GTGAAGAGTGTCGGATTGGCT-3’

NEFH R: 5’-ACACAGAGGGAATTTTGGGGA-3’

Brach_F: 5’-TATGAGCCTCGAATCCACATAGT-3’

Brach_R: 5’-CCTCGTTCTGATAAGCAGTCAC-3’

Myf5 F: 5’-CTGCCAGTTCTCACCTTCTGA-3’

Myf5 R: 5’-AACTCGTCCCCAAATTCACCC-3’

c-actin F: 5’-GTACCCTGGTATTGCTGATCG-3’

c-actin R: 5’-CCTCATCGTACTCTTGCTTGCT-3’

## Results

### Purification and assessment of highly pluripotent human embryonic stem cells

We aimed to develop a cell culture system that can detect teratological impacts on human embryonic cells so we chose to use hESCs as they can be coaxed to differentiate into a wide variety of endodermal, ectodermal, and mesodermal differentiated cell types. However, working with pluripotent cells can be challenging since hESCs can undergo undesired alterations during cell culture that can reduce their pluripotency [20, 22]. Thus we devised a battery of pluripotency assays based on both pluripotency markers and functional assessments of pluripotency so that hESC cultures of validated quality could be used. We also developed strategies to purify highly pluripotent cells from initial hESC cultures for subsequent use in teratology assays.

**Fig 1:**
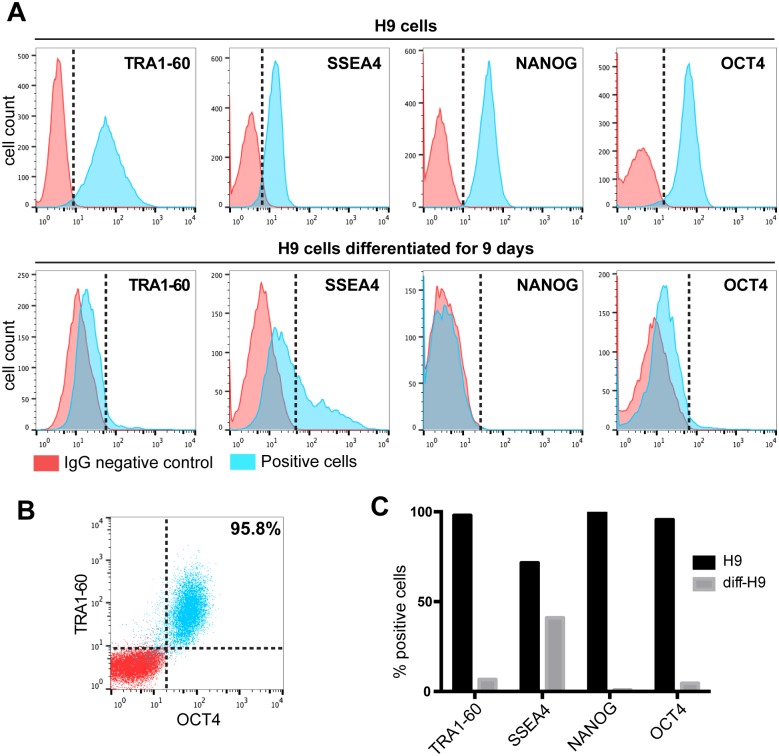
Simultaneous detection of four pluripotency markers by flow cytometry. (A) Flow cytometric analysis of permeablized H9 hESCs. Cells were exposed to antibodies against TRA1-60, SSEA4, NANOG and OCT4. IgG antibody was used as negative control. Top: Cytometry profiles of undifferentiated H9 cells. Bottom: Cytometry histograms of H9 cells 9 days after removal of bFGF. (B) Flow cytometric analysis of undifferentiated H9 cells for the co-stained for TRA1-60 and OCT4. (C) Quantification of positive cells for TRA1-60, SSEA4, NANOG and OCT4 in comparison to IgG control for both undifferentiated and differentiated H9 cells.

We first assessed cultures of hESCs for 4 key markers of pluripotency. These consisted of two cell surface markers (TRA-60 and SSEA4) and two nuclear markers (OCT4 and NANOG) which were chosen because they are master regulatory transcription factors necessary for the maintenance of the pluripotent state in hESCs and induced pluripotent stem cells human (hiPSCs). Though we routinely stain such cells for these markers by immunofluorescence, we wished to quantitatively assess these markers using flow cytometry to more rigorously assess the expression of pluripotency-associated markers in our starter cell cultures (Fig 1A). To achieve this goal, we dissociated cultures to single cells, fixed, and permeablized the cells so that nuclear the nuclear markers (OCT4 and NANOG) are accessible to antibodies, while preserving the surface markers TRA1-60 and SSEA4. We found that human hESC line H9 (WA09) is strongly positive for all 4 markers as compared to control cells stained with non-specific IgG. As expected, undifferentiated H9 cells were positive for TRA1-60 (>95%), SSEA4 (>75%), NANOG (>95%) and OCT4 (>95%) when compared to IgG stained control cells (Fig 1A). To functionally investigate the suitability of these markers, H9 cells were differentiated by removing basic FGF for 9 days, after which only few cells remained positive for TRA1-60 (<10%), NANOG (<5%) and OCT4 (<10%) (Fig 1A). However, we found that SSEA4 is a less robust marker of pluripotency since after 9 days of differentiation about 50% of cells retained SSEA4 expression. We also performed two channel flow cytometry on undifferentiated H9 cells and found that 96% of H9 cells were doubly positive for pluripotency markers TRA1-60 and OCT4 (Fig 1B). We also quantitated the decreases in pluripotency marker expression caused by removal of basic FGF from the medium for 9 days. All markers declined robustly upon differentiation, except SSEA4, whose expression was the slowest to respond to removal of basic FGF (Fig 1C). These studies revealed that TRA1-60 is a superior surface marker of pluripotency as compared to SSEA4, and that both nuclear transcription factors (NANOG and OCT4) were expressed only in the undifferentiated cells.

To further assess the behaviour of SSEA4 in pluripotent cell cultures, we noticed that larger hESC colonies at passage >50 exhibit two patterns of SSEA4 marker expression (S1A Fig). Using immunofluorescence, we found that cells at colony peripheries were highly positive for SSEA4, while cells more central to the colony have lower levels of SSEA4. We then microdissected colonies to isolate peripheral cells (SSEA4 HIGH) and central cells (SSEA4 LOW) and initiated new colonies from the two microdissected subpopulations. In both cases, colonies arose that still exhibited SSEA4 HIGH cells on their peripheries, suggesting an intrinsic regulation of SSEA4 expression that is a function of the position of cells within hESC colonies (S1B Fig). To further investigate this observation, we performed flow cytometry on higher passage cells and found two peaks (S1C Fig), corresponding to SSEA4 HIGH and SSEA4 LOW cells. To further investigate molecular differences between both populations, we isolated SSEA4 HIGH and SSEA4 Low cells using magnetic beads cell purification to positively select for SSEA4 expression cells (S1D Fig). We isolated total RNA from SSEA4 HIGH and LOW cells and performed semi-quantitative reverse transcriptase PCR (RT-PCR) and found that both SSEA4 HIGH and LOW cells still exhibited similar expression levels of OCT4, NANOG, REX1, and hTERT (S1C Fig). We also made standard (random sized) EBs from SSEA4 LOW and HIGH cells, and found that EBs derived form both cell populations robustly expressed mesodermal or ectodermal genes after 17 days of EB differentiation (S1D Fig). We conclude that TRA1-60 is a superior cell surface marker of pluripotency as compared to SSEA4. In addition to the above, we wished to perform functional tests of pluripotency by differentiating cells to ectoderm, endoderm, and mesoderm lineages. We used a kit containing supplements and growth factors allowing the directed differentiation into the three germinal layers in 4 days and the staining of SOX17 (endoderm), OTX2 (ectoderm) and BRACHYURY (mesoderm) (RnD Systems; cat. no. SC027). We found that early passage H9 cells can readily form cells of all three germ layers by this functional test (data not shown).

### Development a standardized and quantitative human EB cell culture system suitable for high-content imaging

**Fig 2:**
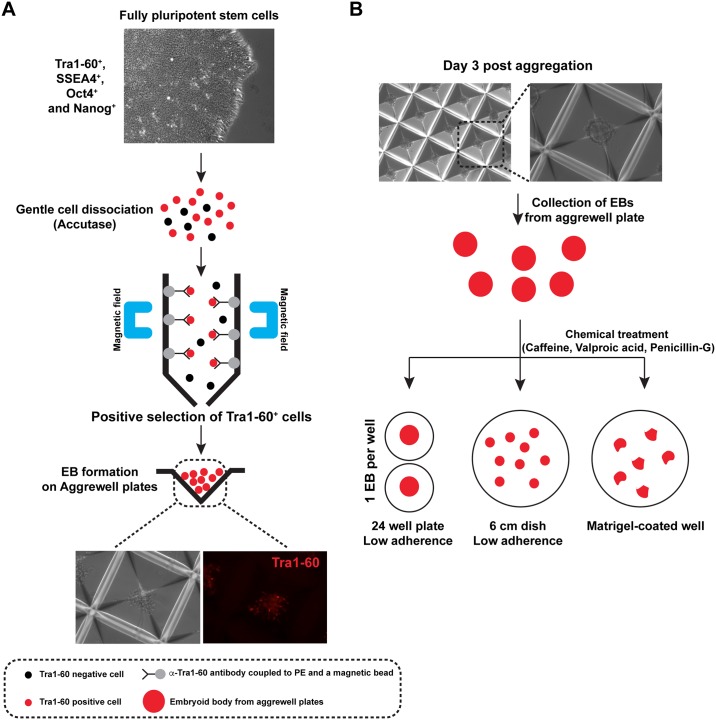
Outline of human quantitative embryoid body (hqEB) system strategies. (A) Preparation of spheroids from TRA1-60+ magnetically purified hESCs. Low passage H9 cells were dissociated to single cells by Accutase treatment, then TRA1-60+ cells were magnetically purified. These were then plated at known density into Aggrewell plates to from spheroids of uniform size. (B) Alternative plating and differentiation approaches. Three methods were assessed: (1) Spheroids were deposited individually into cell culture plate wells, and imaged every 3 days. (2). Pools of EBs are plated into 6 well dishes under non-adherent conditions, imaged, quantitated for size. (3) Spheroids are attached onto Matrigel coated plates and allowed to proliferate and spread under adherent conditions.

hESCs and EBs derived from them have been used as cell culture models to assess toxicological responses to exogenous compounds, but most of these studies have used mouse ES cells and EBs of random size and shape. Though these past approaches have been useful, we wished to develop a highly standardized and quantitative method to detect and analyze teratogens that could be adapted to high-content imaging strategies using mass-produced human EBs of uniform size and shape. One design consideration for us was to insure that only the very best (most highly pluripotent) cells are used to form EBs. We therefore made use of our findings outlined in the section above, and utilized early passage (passage 19) H9 hESCs for this purpose. Furthermore, we found it prudent to dissociate hESC colonies to single cells using accutase-mediated disaggregation to single cells followed by purification of TRA1-60 positive cells by magnetic bead selection (Fig 2A), so that we could be sure that only highly purified populations of hESCs would be used for the initiation of EB cultures. Though we used only highly pluripotent low passage H9 hESCs, we decided to routinely purify TRA1-60 positive cells by this method to initiate all subsequent EB cultures. This strategy insures that starting cell populations used to initiate EB formation are devoid of any contaminating differentiated cells, and this approach also provides a good measure of quality control of the cells used to initiate EB culture.

**Fig 3:**
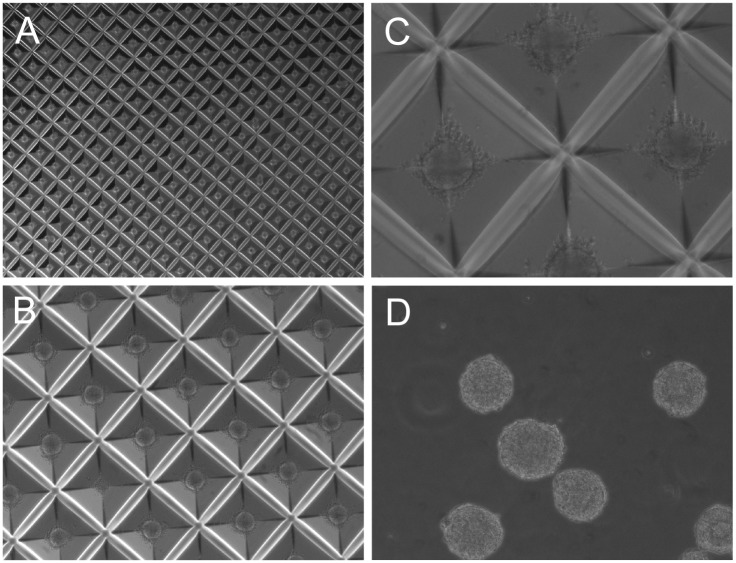
Representative images of hESC spheroids prepared from TRA1-60+ purified cells. Spheroids containing uniform numbers of cells were prepared in Aggrewell plates using TRA1-60^+^ cells. (A) Aggrewell plate containing spheroids aggregated for 3 days (1.25X magnification). (B) Aggrewell plate containing spheroids aggregated for 3 days (4X magnification). (C) Aggrewell plate containing spheroids aggregated for 3 days (10 X magnification). (D) Spheroids imaged immediately after aggregation.

We also wished to mass-produce EBs of initial uniform size to make the EB cultures uniform and synchronous in their growth and differentiation. To achieve this goal, we plated disaggregated and TRA1-60 positive (magnetically purified) cells into Aggrewell plates (Stem Cell Technologies) at an initial seeding density of 2x10^5^ cells per ml in EB culture medium (Stem Cell Technologies). The pyramidal wells of Aggrewell plates are hydrophobic and prevent cellular adhesion to the substratum. We found that after 3 days of aggregation under these conditions, we could produce about 4000 spherical aggregates (spheroids) of hESCs, which were used to initiate EB culture. (Fig 2B). Using these approaches, we were able to produce thousands of uniformly sized human EBs from cultures of hESC line H9 using starting cell populations that are highly uniform and pluripotent, as the cells were positively purified using magnetic beads that bind TRA1-60^+^ pluripotent cells (Fig 3). Once we developed the capability to mass-produce uniform spheroids, we wished to explore alternative means of EB culture and quantification to render the system more versatile of teratogen testing. Uniformly sized and spherical day 0 EBs (spheroids) were harvested from Aggrewell plates, and further cultured in three different ways: First, we deposited individual day 0 EBs into individual wells of low adherence multi-well plates containing differentiation medium using a 200 μl-tip cut at the extremity. The individually plated EBs were allowed to grow and differentiate for an additional 12 days, and since single EBs were deposited into each well, we could monitor the growth and behavior of individual EBs as a function of time. Secondly, we plated pools consisting of hundreds of uniform day 0 spheroids into low adherence 6 cm dishes and allowed them to differentiate for 12 days. This strategy allowed us to monitor EB growth on populations of EBs so that statistically rigorous assessments of mean EB size (growth) could be obtained. Lastly, we plated EBs into Matrigel-coated wells. This strategy allows EBs to adhere to the cell culture substrate, which allows cells to proliferate and grow outward from the core of attached EBs. These combined approaches allowed us to quantitatively assess the growth of EBs under 3 conditions, using cell culture strategies that could easily be adapted for high-content image analysis.

### Performance of the quantitative human EB model with selected teratogens

To assess the sensitivity and accuracy of our EB system (hereafter called the human quantitative embryoid body teratology model system, hqEB system, outlined in Fig 2) we chose to use three compounds known to have teratogenic activities. We chose to study caffeine (a weak teratogen), penicillin-G (a moderate teratogen), and Valproic acid (VPA, highly teratogenic). Caffeine has been studied in the context of embryonic development and was reported to impair normal chick neurodevelopment especially affecting eye formation [23, 24]. Treatment of pluripotent stem cells with caffeine also dysregulates the transcription of important early differentiation genes [19]. Penicillin-G was implicated in kidney development defects in the rat and has been widely studied for its developmental neurotoxicity [25, 26]. Neural teratogenicity has been observed after treatment of human embryonic stem cells with VPA [27]. VPA exposure during pregnancy can cause ear malformation [28]. Our goal was to determine if the hqEB system can robustly and quantitatively assess teratogenicity based on the potential effects of these reference teratogens in our newly developed system.

### Determination of caffeine, penicillin-G and valproic acid sub-cytotoxic concentrations and teratogen assay dosing

In principle, EB growth kinetics could be affected by either simple cellular cytotoxicity, or due to teratogenic effects that alter cellular proliferation and differentiation. Since we were more interested in teratogenicity as opposed to overt cytotoxicity, we performed dose-response cytotoxicity assays to determine the LC_50_ for each of the three compounds. We first determined by dose response the cytotoxicity of the drugs at five concentrations in two human fibroblast cell lines: I90-83 (also called IMR-90) and I91S-04 (also called IMR-91) (S2A Fig). The results indicated that both fibroblast cell lines had similar LC_50_ values upon exposure to each teratogen. This dosing data allowed us to choose teratogen concentrations for use in the hqEB system that do not elicit overt cytotoxic effects. The concentrations chosen for hqEB analyses in this study were 0.26 mM, 2.8 mM and 1 mM for caffeine, penicillin-G and VPA respectively. These concentrations are 10-fold or more below the LC_50_ for each compound. To further control for potential cytotoxicity of the compounds at these concentrations, we treated hESC H9 cells for 72 hours at these sub-cytotoxic concentrations. We found no significant impact on hESC cell colony growth or morphology after 72 hours of treatment (S2B Fig).

### Individual EB monitoring

**Fig 4:**
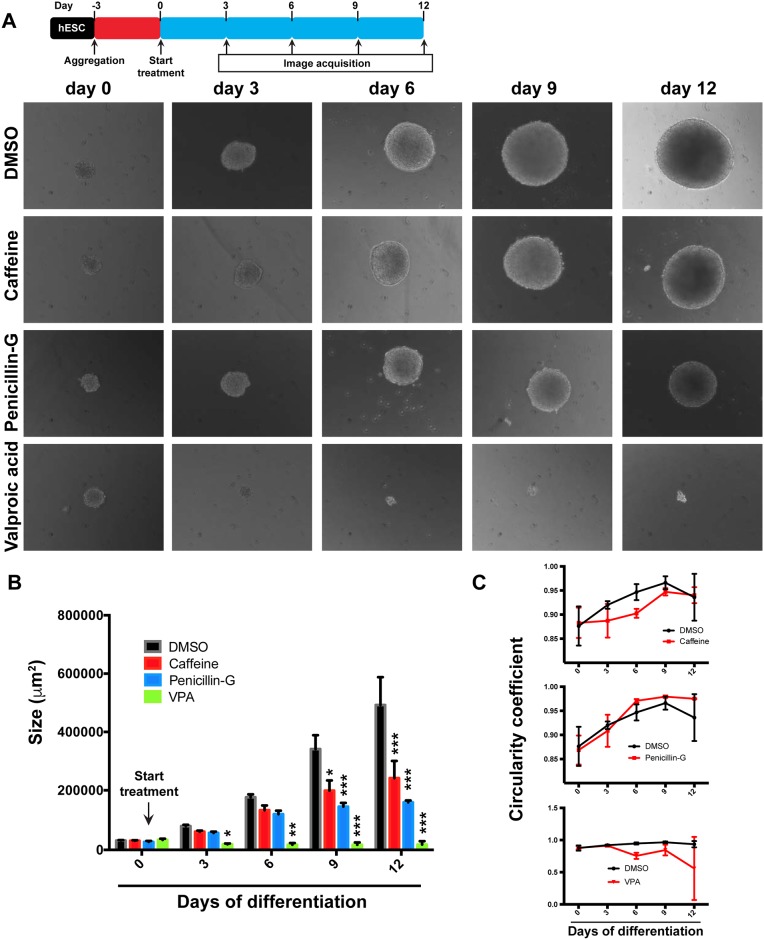
Consequences of teratogen exposure on individually cultured EBs. (A) H9 hESCs were dissociated with aAccutase, formed into spheroids, and plated into individual wells under non-adherent conditions (day 0). Media was changed to EB differentiation medium and individual EBs were imaged microscopically every 3 days for a total of 12 days of differentiation. Individual EBs were treated with vehicle only (DMSO), Caffeine, Penicillin-G, and Valproic acid and images were acquired after 0, 3, 6, 9, and 12 days of differentiation. (B) EBs were quantitated by measuring the apparent size (μm^2^) of individual embryoid bodies after treatment with DMSO, Caffeine, Penicillin-G or Valproic acid. Results shown indicate the means and standard deviations by quantitatively imaging a cohort of 3 independently cultured EBs each tested with each of the 3 compounds. (*P<0.05; **P<0.01; ***P<0.001 as compared to DMSO). (C) Shape quantification expressed by the circularity coefficient of individual EBs treated with DMSO, Caffeine, Penicillin-G or Valproic acid. Results shown indicate the means and standard deviations obtained by quantitatively imaging a cohort of 3 independently cultured EBs each tested with each of the 3 compounds.

We first investigated the use of individually cultured, non-adherent EBs exposed to the reference teratogens. First, we prepared hESC spheroids using Aggrewell plates as described above, using magnetically purified TRA1-60^+^ cells to initiate spheroid formation. After 3 days of aggregation (hereafter designated as day 0), spheroids of uniform size and shape were deposited individually into single wells of low adherence 24-well plates, the media was changed from mTeSR1 to EB differentiation medium, and 3 reference teratogens were added at the established sub-cytotoxic concentrations. Four individual EBs were grown in the presence of each teratogen (quadruplicate). We analyzed the growth and shape of individual EBs by microscopy every 3 days for a total of 12 days in differentiation medium. (Fig 4A). Control EBs that were not treated with teratogens exhibited consistent growth over the course of the experiment. In contrast, exposure to VPA greatly curtailed the growth of EBs. After only 3 days of teratogen exposure, we observed that the growth of VPA-treated EBs was arrested, and this failure to grow persisted throughout the course of the experiment (Fig 4A and 4B). In the case of caffeine (weakly teratogenic), we observed a modest but detectable effect on EB growth, which became apparent and statistically significant at days 9 and 12. The effect of penicillin-G (moderately teratogenic) was stronger than that of caffeine, but less severe than VPA. EBs treated with Penicillin-G failed to increase significantly in size after 6 days of treatment (Fig 4A and 4B). We used microscopy imaging software to quantitatively assess impacts on the shape of EBs. To do this, we calculated the circularity coefficient for each time point (expressed as circularity = 4π(area/perimeter^2^)). This measure showed that shape alterations can also be detected in the hqEB system. We found subtle effects on EB circularity using this approach in the case of caffeine (Fig 4C). Together these results show that teratogenic effects can be readily detected by the hqEB system. We note that these findings suggest that the hqEB system could easily be adapted to utilize automated high-content imaging solutions in future larger scale applications.

### Additional analyses of teratogen-exposed EBs

**Fig 5:**
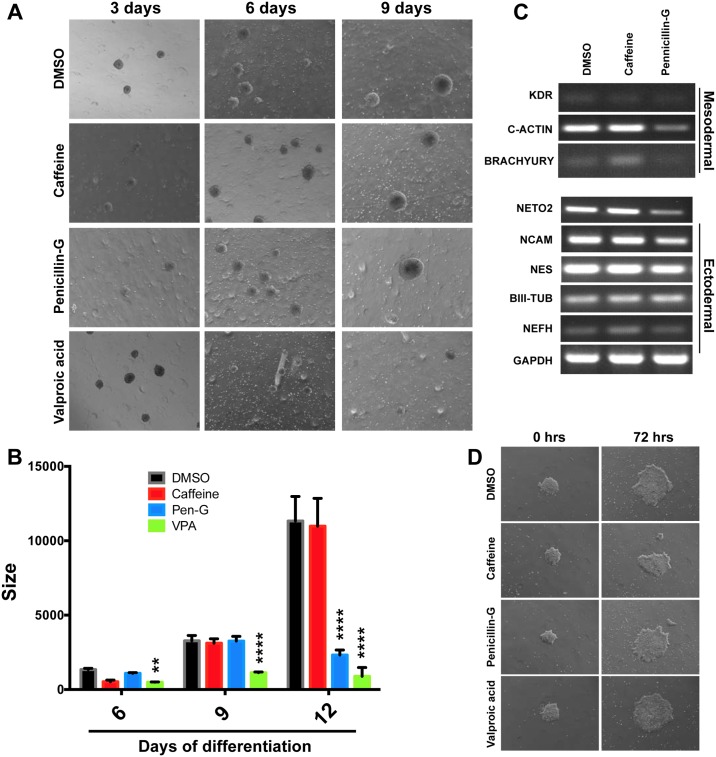
Monitoring EB growth in pooled populations of EBs grown under non-adherent conditions. (A) Representative images of EBs grown in the presence of DMSO (vehicle only), Caffeine, Penicillin-G and Valproic acid. (B) Size quantification of EB populations treated with DMSO, Caffeine, Penicillin-G or Valproic acid. Results are the means +/- standard deviation. (n = 17 to 69 independent EBs after 3, 6, and 9 days of culture in the presence of teratogens (*P<0.05; **P<0.01; ***P<0.001 as compared to DMSO). (C) Analysis of mRNA expression of differentiation markers from EBs treated with DMSO, Caffeine or Penicillin-G over the course of 12 days of EB differentiation. RT-PCR performed to detect mesodermal (KDR, C-ACTIN and BRACHYURY) and ectodermal mRNAs (NETO2, NCAM, NES, BIII-TUB and NEFH). GAPDH is used as loading control. (D) EB spreading assay performed on attached EBs cultured on Matrigel (0 hours) and treated with DMSO, Caffeine, Penicillin-G or Valproic acid for 72 hours in differentiation medium.

In addition to individual EB monitoring, we wanted to see if similar results could be obtained by analyzing pools of EBs exposed to teratogens. To do this, dishes containing at least 100 unattached EBs of the same size were treated with the three compounds for 12 days and analyzed by microscopy every three days (Fig 5A). The diameters and growth kinetics of EBs were consistent with the previous assay, and VPA teratogenicity was again detected after only three days of teratogen exposure, while EBs treated with Penicillin-G stopped growth by 9 days after aggregation (Fig 5B). Using this approach, caffeine treatment exhibited little or no effect on EB growth (Fig 5B). EBs were then analyzed by qRT-PCR for induction of selected germ layer gene expression (Fig 5C). We found that mesoderm and ectoderm genes were induced and robustly expressed by day 12. We found that caffeine treatment had no major effects on expression of these genes, while penicillin-G treatment had some effect on expression of these genes. Lastly, we wished to see if we could track compound teratogenicity in culture conditions in which spheroids are allowed to attach to the culture substratum. We therefore aggregated H9 hESCs for 3 days and plated the spheroids onto Matrigel-coated plates concurrently with change to differentiation medium. As expected, we noted outgrowths of differentiated cells the spread out from the centers originally formed at the sites of EB attachment. The outgrowths were irregular in shape, and after 72 h of treatment with the three compounds, no phenotypic differences were observed (Fig 5D). Our results indicate that monitoring individual EBs grown under non-adherent conditions constituted an approach that robustly and quantitatively detected teratogenic effects. In contrast, we found the adherent EB strategy less useful, because outgrowth area and shape are more variable.

## Discussion

We developed and refined a human EB system that utilizes highly characterized cells of demonstrated pluripotency in such a way that the effects of teratogens can be evaluated and quantitated by numerical descriptors with statistical accuracy. Since teratogens impact the course of directed cellular differentiation during embryogenesis, we strived for utilizing only the very highest quality of cells to initiate EB cultures. To this end, we assessed hESCs rigorously for their pluripotency-associated marker expression. We found that TRA1-60 is a superior surface pluripotency marker as compared to SSEA4. Furthermore, we wished to initiate our EB cultures with spherical aggregates of hESCs, which we found could be mass-produced in aggrewell plates. To do this, we faced a challenge in that suspensions of single cells are required in order to seed aggrewell plates. This is complicated by the fact that cell-cell contacts (mediated by E-Cadherins in hESCs) are required to maintain the pluripotent state of hESCS. We therefore developed a strategy whereby feeder cell-free starter cultures of hESCs are disaggregated to single cells by Accutase treatment. The singularized cells were maintained in the presence of ROCK inhibitor, which can stabilize the maintenance of pluripotency of single hESCs during the disaggregation of hESC colonies to single cells. We also immunologically purified TRA1-60^+^ cells using magnetic beads and used these to seed aggrewell plates to prepare large numbers of uniform spheroids that could in turn be used to initiate EB cultures. We found that we can prepare large numbers of EBs by this method, which are of initial uniform size, and whose growth and proliferation occur synchronously. We found that by simply monitoring a cohort of EBs treated with teratogens (at doses that are sub-cytotoxic), that we could observe defects in EB growth characteristics that could be quantified precisely. We were able to reproducibly and quantitatively detect effects on EB development using a panel of three reference teratogens. This report builds on previous successes with human EBs, but our system makes use of highly standardized EBs of known pluripotency which are of standardized and uniform size and pluripotency prior to differentiation in the presence of potentially teratogenic compounds.

The hqEB system has features that make it attractive for future assessments of teratogen action. First, our system utilizes relevant human cells of embryonic cellular identity (hESCs), which model the inner cell mass of blastocyst-stage preimplantation embryos. Furthermore, EBs differentiate in vivo to form primitive cells of ectodermal, endodermal, and mesodermal lineages. Also, hESCs and EBs derived from them contain intact complete genomes, and offer obvious advantages as compared to immortalized cell lines. We also initiated our EBs with known numbers using TRA1-60^+^ immunopurified hESC starter cells and found that EB growth (and the impacts of teratogen exposure) can be monitored continuously and assessed quantitatively. The hqEB system is of much higher throughput than costly animal experiments. Though these are useful features, the EB system also has limitations. Firstly, metabolites of parent compounds may be responsible for teratogenicity *in vivo*, and the EB system presented here utilizes relevant embryonic cells (the targets of teratogens) but these cell types cannot metabolize parent compounds. Thus the hqEB system is at present not suited to the assessment of unknown metabolites of parent compounds. In addition, EB systems in general contain early embryonic cells, and therefore later developmental effects cannot easily be modeled with such cells. Therefore, future uses of the hqEB system and its future iterations should be of good use, but it is unlikely that its use will fully supplant the use of animals studies, which could better detect subtle and late-term impacts on embryo health (though with the caveat that rodent embryonic development is clearly not the same as human embryonic development).

In the future, it should be relatively straight forward to adapt the hqEB system to automated high content imaging, since individual EBs can be cultured individually in multi-well plates and assessed periodically by automated cell culture microscopy approaches and associated image acquisition and analysis software. We also note that our uniform EBs could also be cultured in multiplexed microfluidics devices in which continuous perfusion and compound dosing can be performed. It is also noteworthy that complex mixtures (including drinking water samples) could readily be assessed by these approaches. For these reasons, we suspect that improved human EB methods such as those described here will gain added importance in future large scale assays of teratogens. Furthermore, the system would be useful during safety assessment stages of development of new pharmaceuticals. The approaches described in this study should allow the assessment of hundreds of potential teratogens since the assay is based on morphological assays of EBs that are initially uniform, develop synchronously, and that can be assessed at intervals over the course of differentiation. Furthermore, our approaches are readily adaptable to microfluidics and high-content imaging. Once prospective teratogens are identified by these expedient means, it should then be possible to perform more rigorous studies of teratogenic mechanisms by investigating the impacts of compounds on the cellular composition of impacted EBs (including their relative content and identities of cells of ectodermal, endodermal, and mesodermal origin) as well as detailed analyses of altered gene and protein expression, which collectively, should shed light on specific developmental pathways impacted by specific teratogens.

## Supporting information

S1 Fig Analysis of pluripotency marker expression in hESC line H9. (A) High passage H9 cell colony live stained with α-SSEA4 antibody coupled to PE. i and ii (insets) show colony regions expressing high (colony peripheries) and low (colony interior) levels of SSEA4 respectively. (B) Microdissection of H9 cell colonies to isolate peripheral cells (SSEA4 HIGH) and central cells (SSEA4 LOW). Bottom: representative colonies initiated with SSEA4 HIGH and LOW dissected cells after 10 passages. (C) Flow cytometry analysis of high passage H9 cells for the stem cell marker SSEA4. Note the presence of two populations of cells expressing different levels of SSEA4 as compared to IgG negative control. H9 colonies were dissociated to single cells and incubated with an anti-SSEA4 antibody coupled to magnetic beads. Immunopurified cells express high levels of SSEA4. (D) Pluripotency marker expression analysis on hESC line H9 sorted for TRA1-60 or SSEA4 (low and high expression). RT-PCR was used to detect OCT4, NANOG, REX1 and hTERT mRNAs and GAPDH was used as loading control. To determine if the expression of SSEA4 status impacts differentiation, H9 EBs were made from hESCs expressing high or low levels of SSEA4 and then differentiated for 17 days. RT-PCR performed on mRNA isolated from the EBs and assessed for expression of KDR, C-ACTIN, BRACHYURY (mesoderm) and NETO2, NCAM, NES, BIII-TUB and NEFH (ectoderm). GAPDH is used as an internal loading control.
(TIF)

S2 Fig Determination of sub-cytotoxic compound doses. (A) Dose-response curves of teratogens two human fibroblast cell lines (I90-83 and I91S-04) exposed to caffeine, penicillin-G, and Valproic acid. Red arrow indicates the concentration chosen for the hqEB system. (n = 3 independent experiments). (B) Representative images of hESC line H9 treated with DMSO or Valproic acid for 24, 48 and 72 hours. Insets show higher magnification images of cellular morphology within colonies.
(TIF)
