# Supplementary figures and images for: A standardized human embryoid body platform for the detection and analysis of teratogens
Source: PLoS One. 2017 Feb 9;12(2):e0171101. doi: 10.1371/journal.pone.0171101 (PMC5300235; doi:10.1371/journal.pone.0171101)

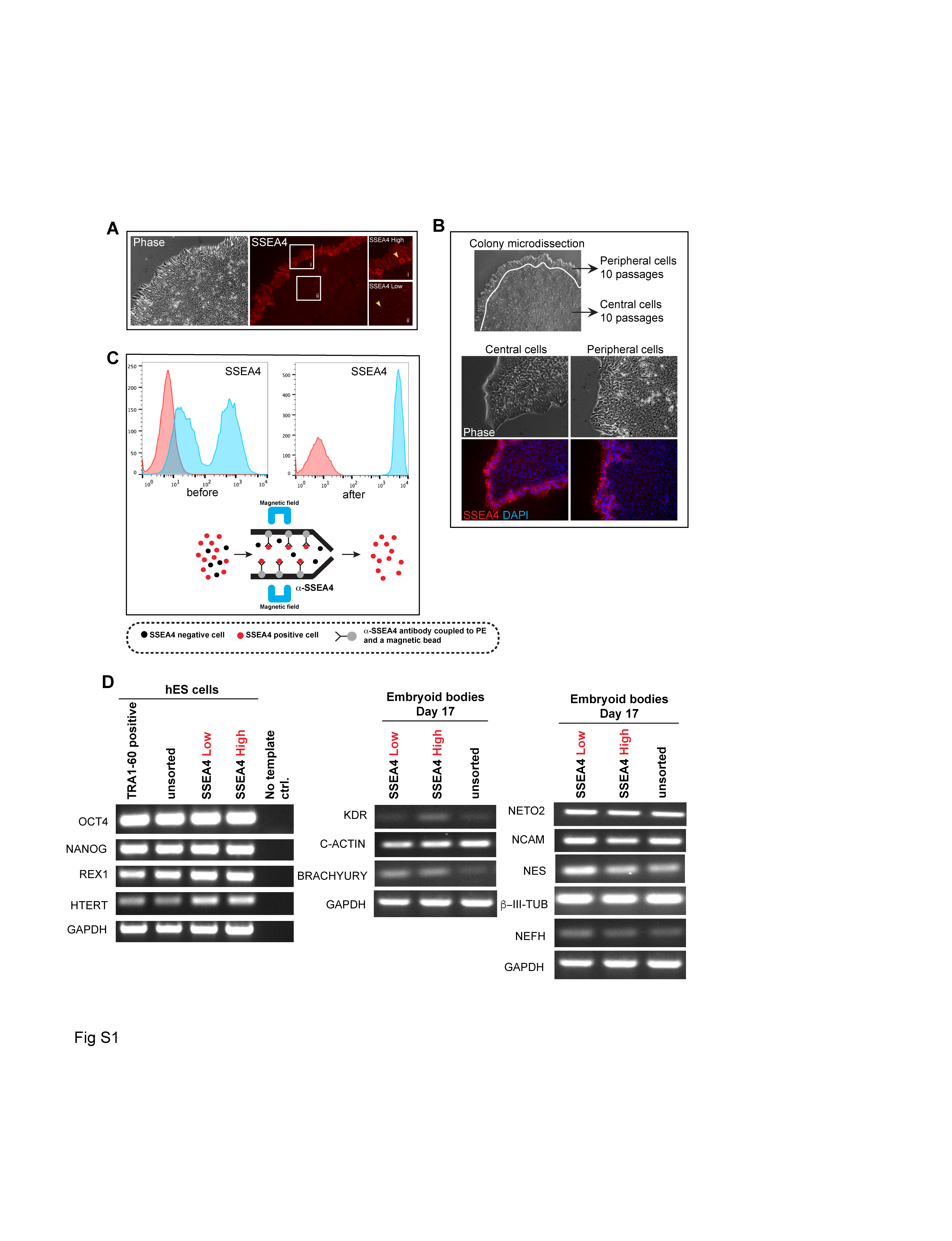

Supplement: S1 Fig — (A) High passage H9 cell colony live stained with α-SSEA4 antibody coupled to PE. i and ii (insets) show colony regions expressing high (colony peripheries) and low (colony interior) levels of SSEA4 respectively. (B) Microdissection of H9 cell colonies to isolate peripheral cells (SSEA4 HIGH) and central cells (SSEA4 LOW). Bottom: representative colonies initiated with SSEA4 HIGH and LOW dissected cells after 10 passages. (C) Flow cytometry analysis of high passage H9 cells for the stem cell marker SSEA4. Note the presence of two populations of cells expressing different levels of SSEA4 as compared to IgG negative control. H9 colonies were dissociated to single cells and incubated with an anti-SSEA4 antibody coupled to magnetic beads. Immunopurified cells express high levels of SSEA4. (D) Pluripotency marker expression analysis on hESC line H9 sorted for TRA1-60 or SSEA4 (low and high expression). RT-PCR was used to detect OCT4, NANOG, REX1 and hTERT mRNAs and GAPDH was used as loading control. To determine if the expression of SSEA4 status impacts differentiation, H9 EBs were made from hESCs expressing high or low levels of SSEA4 and then differentiated for 17 days. RT-PCR performed on mRNA isolated from the EBs and assessed for expression of KDR, C-ACTIN, BRACHYURY (mesoderm) and NETO2, NCAM, NES, BIII-TUB and NEFH (ectoderm). GAPDH is used as an internal loading control. (TIF) [file pone.0171101.s001.tif]

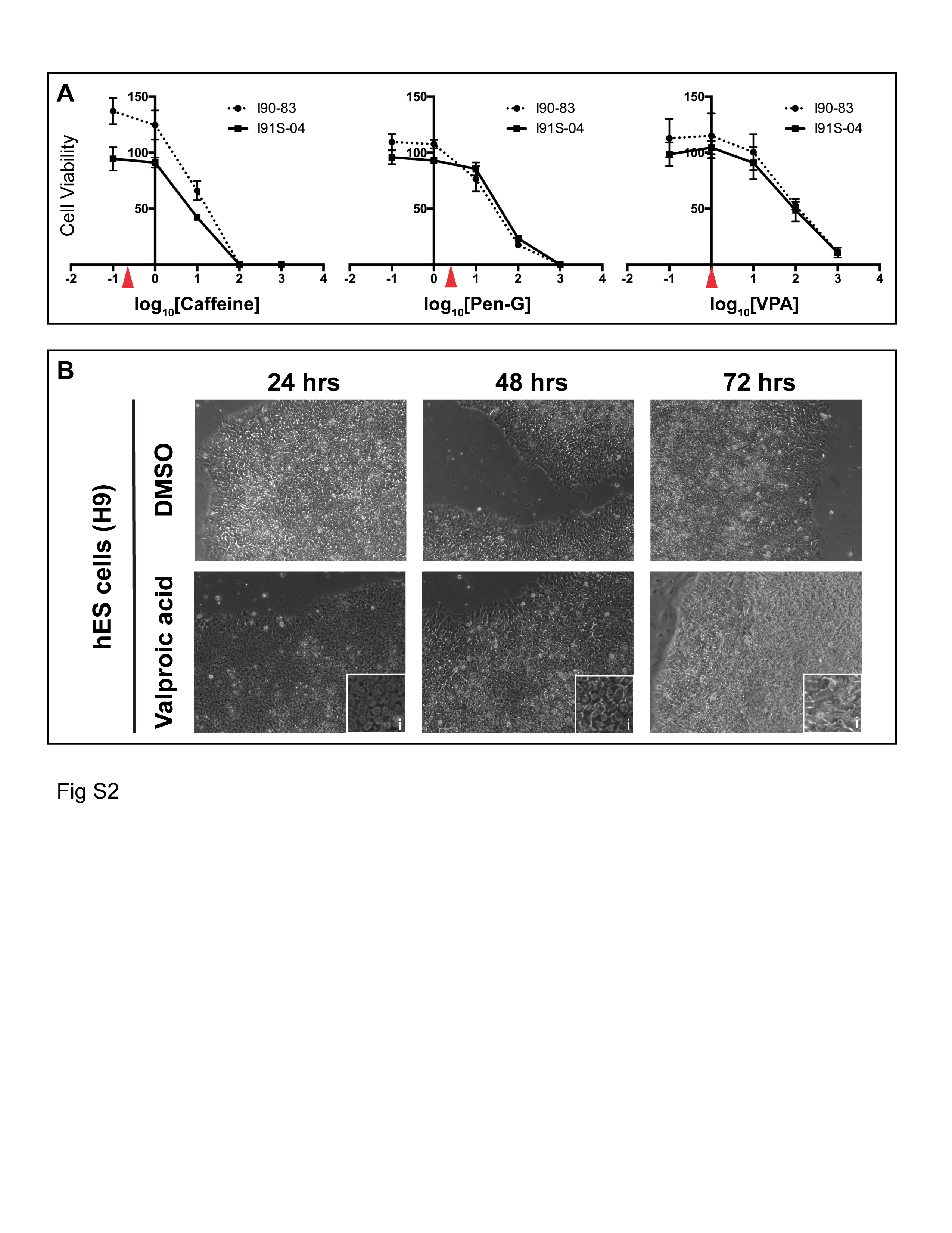

Supplement: S2 Fig — (A) Dose-response curves of teratogens two human fibroblast cell lines (I90-83 and I91S-04) exposed to caffeine, penicillin-G, and Valproic acid. Red arrow indicates the concentration chosen for the hqEB system. (n = 3 independent experiments). (B) Representative images of hESC line H9 treated with DMSO or Valproic acid for 24, 48 and 72 hours. Insets show higher magnification images of cellular morphology within colonies. (TIF) [file pone.0171101.s002.tif]
